# 

**Published:** 1899-05-06

**Authors:** 


### NOTICE TO CORRESPONDENTS.

All MS., Letters, Books for Review, and other matters intended for the Editor should be addressed The Editor, "The Hospital," The Hospital Building, 28 & 29, Southampton Street, Strand, W.C.

The Editor cannot undertake to return rejected MS., even when accompanied by stamped directed envelope.

**Notice to Advertisers and Subscribers.**

All Advertisements, Orders for Copies of The Hospital, and Business Communications should be addressed to **THE MANAGER (Not to the Editor),** The Hospital, The Hospital Building, 28 & 29, Southampton Street, Strand, London, W.C.

The Cost of Subscription, post free, is as follows:—Per week, 2½d.; per quarter, 2s. 8d.; per half-year, 5s. 3d.; per annum, 10s. 6d. Foreign:—Per annum, 15s. 2d., payable, in all cases, in advance.

**NOTICE.—Vol. XXV. of "The Hospital" (October, 1898, to March, 1899), in handsome cloth binding, is now on sale at the Office of this Journal, price 7s. 6d. Cases for binding the Numbers contained in this Volume 1s. 6d. Index to Vol. XXV., 2½d., post free.**

**The Monthly Part for May is now ready. Price 6d., by post 8d.**

### BOOKS RECEIVED.

Cassell and Co.

"Hygiene and Public Health." A Manual by B. Arthur Whitelegge, M.D.

"Materia Medica and Therapeutics." By J. Mitchell Bruce, M.D.

### SCRAPS AND GLEANINGS.

At the annual meeting of the Norfolk and Norwich Hospital it was stated that £300 was still needed to fit up the operation theatre in proper modern style.

Last year the actual income available by the Norfolk and Norwich Hospital for current work was £8,678, while the expenditure amounted to £10,384. To meet this deficit the Board of Management had to sell out £2,700 of their invested funds.

The Derbyshire Royal Infirmary have now made the rule hitherto not in use at that institution that the number of visitors to each patient is to be limited. This has been rendered necessary seeing that so many as seven or eight hundred visitors have entered the wards to see patients on visiting days.

After some discussion by the Council of the City of Lincoln it has been resolved to provide an ambulance conveyance for taking patients to the fever hospital. The objectors to this resolution based their opposition on the fact that it was an unnecessary expenditure, as there was no Notification Act in force in that city.

The Duke of Somerset has made a grant in perpetuity to the Totnes Hospital Committee of a site for a permanent hospital at a ground rental of £2 10s. for the first six years and at the rate of £10 per acre per annum afterwards. This generous offer has been gratefully accepted by the Totnes Hospital Committee, who are now to advertise for plans.

At the annual court of governors of the Royal Berkshire Hospital the report of the Special Committee of Inquiry which had been appointed to consider the question of retrenchment was accepted. In this report the Special Committee expressed their opinion based on comparison with other hospitals, that it was advisable that some of the beds should be closed.

It has been resolved by the other districts concerned to appeal against the petition by Meltham against inclusion for isolation hospital purposes within the district of the Holme and Colne Valleys. The appellants base their objections to the petition on the ground that Meltham district is only one-tenth of the whole district, and that as it would be a much more economical arrangement, for various reasons, if Meltham were included, the petition of so small an area should not be granted under the circumstances.

